# Integrative bioinformatics and in vivo validation suggest a potential role of *Cbr4* in alcohol use disorder through modulation of lipid metabolism and treg cell function in the central amygdala

**DOI:** 10.3389/fgene.2026.1829878

**Published:** 2026-07-01

**Authors:** Yao Ge, Yihan Yuan, Min Li, Zhenxia Huang, Chenli Zhang, Caiyu Yu, Jinyu Xu, Siao Zhang, Hongwei Guo, Xu Zhang, Rao Fu, Zhiheng Ren

**Affiliations:** 1 Department of Pathology, School of Basic Medical Sciences, Lanzhou University, Lanzhou, Gansu, China; 2 The First School of Clinical Medicine, Lanzhou University, Lanzhou, Gansu, China; 3 The Second Clinical Medical School, Lanzhou University, Lanzhou, Gansu, China; 4 Department of Pathology, Qingyuan Hospital, Guangzhou Medical University, Qingyuan, Guangdong, China; 5 Department of Neurology, The Second Hospital of Lanzhou University, Lanzhou, Gansu, China; 6 Department of Anatomy, School of Medicine, Shenzhen Campus of Sun Yat-sen University, Sun Yat-sen University, Shenzhen, Guangdong, China

**Keywords:** alcohol use disorder, *Cbr4*, central amygdala, lipid metabolism, mendelian randomization, regulatory T cells

## Abstract

**Background:**

The central amygdala (CeA) is closely associated with the development of alcohol dependence by mediating negative affective and stress responses during alcohol withdrawal. Chronic alcohol disrupts CeA neuroimmune balance and lipid metabolism, accelerating alcohol use disorder (AUD), but the regulatory mechanisms remain unclear. This exploratory study aimed to identify CeA key genes associated with AUD and investigate their potential involvement in mediating these dysregulations.

**Methods:**

Based on the GSE31708 dataset, differential gene analysis, protein-protein interaction (PPI) network, random forest, and LASSO regression were used to screen hub genes. Adult male Long-Evans rats were trained to consume alcohol over 8 weeks; open field test (OFT) and elevated plus maze (EPM) assessed withdrawal-induced anxiety, and RT-qPCR validated candidate genes. CIBERSORT analyzed immune cell composition, co-expression networks delineated molecular interactions, and Mendelian randomization (MR) explored potential genetic associations.

**Results:**

Hub gene *Cbr4* was found to be significantly upregulated in the CeA of AUD subjects. Preliminary *in vivo* validation revealed increased *Cbr4* expression in alcohol-withdrawn rats, and a negative correlation was observed between *Cbr4* expression and anxiety-like behavioral parameters. Mendelian randomization analyses suggest that *Ephx2* -a pivotal molecule in the co-expression network-has a genetic association with *Cbr4* (OR = 1.32, 95% CI = 1.15–1.51, *p* = 0.031). Immune infiltration analysis indicated reduced regulatory T cell (Treg) levels in AUD subjects, with *Cbr4* expression showing a negative correlation with Treg abundance. Multiple Treg subtypes appeared to be associated with a protective effect against AUD (OR = 0.9744–0.9797, *p* = 0.036–0.043).

**Conclusion:**

Our findings suggest that *Cbr4* in the CeA may play a role in the pathological processes of AUD. It may contribute to AUD development through two potential mechanisms: a possible genetic interaction with *Ephx2*, and a negative association with Treg abundance, which may be associated with dysregulation of CeA neuroimmune homeostasis. These preliminary observations provide a basis for further investigation into *Cbr4* as an exploratory molecular indicator for AUD.

## 1 Introduction

Alcohol is one of the most prevalent psychoactive substances worldwide. Chronic alcohol consumption induces extensive and profound alterations in the central nervous system**(** Rehm et al., 2025), which subsequently contribute to the development of various psychiatric symptoms—such as relapse, anxiety, depression, and bipolar disorder (Kranzler, 2023). Drawing on the 2023 dataset published by the World Health Organization (WHO), approximately 3.3 million individuals die worldwide each year as a consequence of harmful alcohol use. This figure corresponds to 5.9% of all global deaths, a statistic that underscores the profound public health burden associated with alcohol use disorder (AUD) (Rodriguez, 2021). A burgeoning literature has demonstrated that prolonged alcohol intake exerts selective modulatory effects on neural circuits underlying addictive behaviors, including the prefrontal cortex–lateral habenula–amygdala circuits (Huang et al., 2026; Song et al., 2026)—a neurobiological mechanism that promotes the onset and progression of AUD. This process encompasses the adaptive remodeling of synaptic plasticity within brain reward, stress, and emotional regulation systems, which ultimately precipitates pathological perturbations in the central nervous system (Roberto et al., 2021).

Among the key brain regions implicated in the onset and progression of AUD, the central amygdala (CeA) has been identified as the nucleus with the strongest etiological association. As a vital regulatory hub in the neurobiology of drug addiction (Melkumyan and Silberman, 2022), aberrant neuroimmune signaling in the CeA disrupts the coordinated function of stress and reward pathways, driving the development of alcohol dependence, increased alcohol intake, and relapse vulnerability (Melkumyan et al., 2025). Collectively, these findings suggest the CeA as a crucial neural substrate for the “stress–addiction” vicious cycle that characterizes AUD (Cruz et al., 2024; Melkumyan et al., 2025). However, significant gaps still exist in our mechanistic understanding of the molecular events that govern these pathological processes within the CeA.

It is well established that genes such as *ADH* (alcohol dehydrogenase) and *ALDH* (aldehyde dehydrogenase) influence the development of AUD by regulating alcohol metabolic processes (Osna et al., 2022; Adasme-Reyes et al., 2024). However, interventions targeting these metabolic pathways demonstrate limited efficacy, benefiting only approximately 30%–40% of AUD patients, and often fail to alleviate comorbid psychiatric symptoms such as anxiety, depression, irritability, and sleep disturbances (Zawilska et al., 2026). Synaptic plasticity in the CeA is closely associated with emotional regulation and drug addiction. This nucleus may harbor novel molecular targets that drive the onset and progression of AUD (Nentwig et al., 2025). Thus, a systematic exploration of potential regulatory genes within the CeA, coupled with the elucidation of their molecular interaction networks, may not only facilitate the discovery of novel neuroregulatory circuitry but also help address critical bottlenecks in mechanistic research on AUD, which in turn may offer substantial scientific value and translational potential for clinical applications.

**FIGURE 1:**
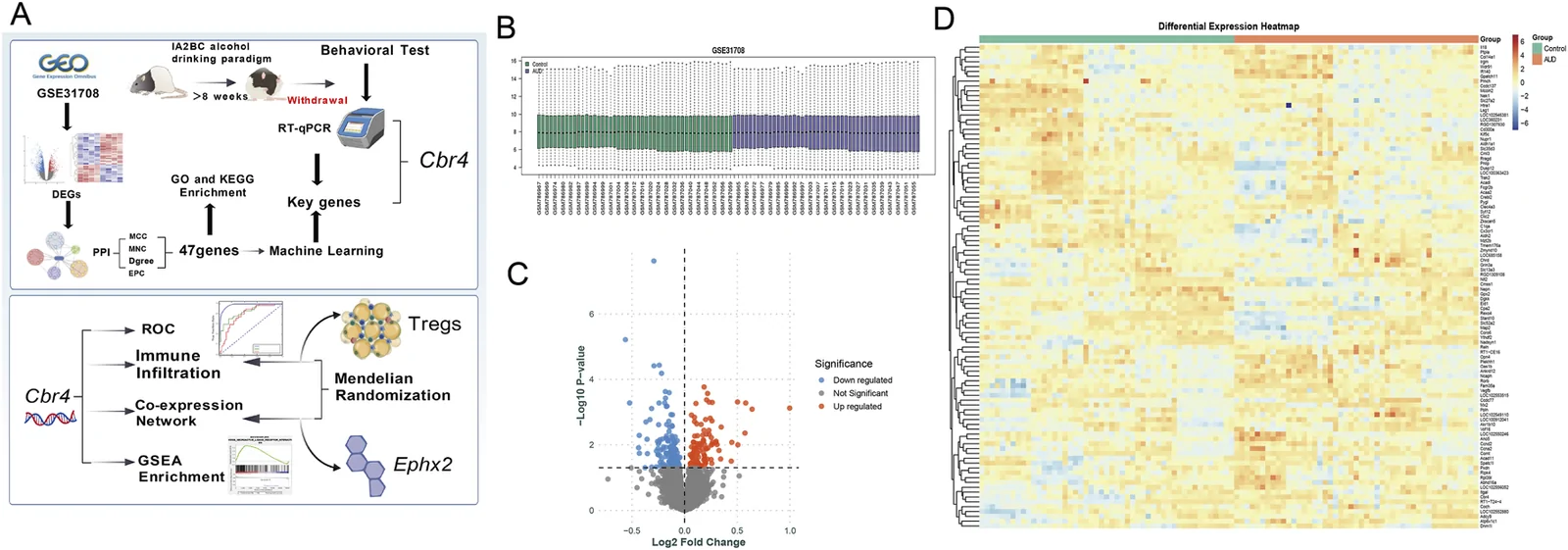
Flowchart of the study design and Identification of differentially expressed genes (DEGs) associated with alcohol use disorder (AUD). **(A)** The study integrated multi-dimensional approaches including bioinformatics analysis (GEO dataset mining, differential gene screening, PPI network construction, machine learning modeling, immune infiltration analysis), *in vivo* validation (IA2BC animal model establishment, behavioral tests, RT-qPCR verification), and causal inference (Mendelian randomization analysis) to identify key regulators and molecular mechanisms of alcohol use disorder (AUD) in the central amygdala (CeA). **(B)** Data normalization of 96 amygdala samples from the GSE31708 dataset. **(C)** Volcano plot of DEGs between the AUD group and control group (*p* < 0.05), with 113 upregulated genes (red dots) and 138 downregulated genes (blue dots). **(D)** Heatmap of the top 50 DEGs ranked by statistical significance (*p* < 0.05), showing distinct expression patterns between the two groups. (DEGs, differentially expressed genes; AUD, alcohol use disorder).

To identify potential regulatory genes in the CeA for AUD, this exploratory study performed bioinformatics analyses using the GSE31708 dataset from the Gene Expression Omnibus (GEO). Through integrated multi-algorithm screening and preliminary animal experiments, *Cbr4* was identified as a candidate gene potentially associated with AUD progression within the CeA. Co-expression network analysis indicated an association between *Cbr4* and *Ephx2*, and these two genes may be jointly involved in the development of lipid metabolism dysregulation. Furthermore, computational immune infiltration analysis and Mendelian randomization (MR) analysis suggested that Tregs, which showed a negative correlation with *Cbr4* expression, exhibited a genetic negative correlation with AUD, indicating a potential protective association. Collectively, these preliminary findings may provide initial clues for exploring the molecular mechanisms underlying AUD, particularly the CeA-related networks involving *Cbr4*, *Ephx2* and local immune responses. The results may also help identify exploratory molecular indicators associated with AUD. The overall workflow of this study is summarized in Figure 1A.

## 2 Materials and methods

### 2.1 Data sources

Gene expression microarray data used in this study were sourced from the Gene Expression Omnibus (GEO) under accession number GSE31708. This dataset contains 96 CeA tissue samples from *Rattus norvegicus*, generated *via* genome-wide expression profiling by microarray technology. Notably, it includes transcriptomic data from five independent selectively bred rat lines with divergent inherent alcohol consumption phenotypes, which were grouped into low alcohol-preferring controls and high alcohol-preferring AUD groups for analysis. Publicly available GWAS datasets for Mendelian Randomization analysis were retrieved from the IEU OpenGWAS database. Detailed information on these datasets is provided in Table 1, 2.

**TABLE 1:** Description of the GEO datasets.

Dataset	Platform	Sample characteristics
GSE31708	GPL1355	The dataset contains 96 central amygdala (CeA) tissue samples from five distinct selectively bred rat lines. Samples were grouped into low alcohol-preferring control lines and high alcohol-preferring AUD model lines based on inherent genetic alcohol preference traits

**TABLE 2:** Description of the GWAS datasets.

OpenGWAS ID	Trait	Population
ebi-a-GCST90001706	BAFF-R on IgD^+^ CD38^-^ naïve B cell	European
ebi-a-GCST90001727	CD19 on IgD^+^ CD38^-^ naïve B cell	European
ebi-a-GCST90001737	CD19 on naïve-mature B cell	European
ebi-a-GCST90001481	Resting CD4 regulatory T cell %CD4 regulatory T cell	European
ebi-a-GCST90001482	Resting CD4 regulatory T cell %CD4^+^ T cell	European
ebi-a-GCST90002069	CD4 on CD39^+^ secreting CD4 regulatory T cell	European
eqtl-a-ENSG00000145439	Cbr4	European
eqtl-a-ENSG00000120915	Ephx2	European
eqtl-a-ENSG00000001036	Fuca2	European
eqtl-a-ENSG00000169313	P2ry12	European
finn-b-AUD	Alcohol use disorder, ICD-based	European

### 2.2 Identification of differentially expressed genes (DEGs)

The “limma” package in R was used to screen for differentially expressed genes (DEGs: experimental design matrices and contrast matrices were constructed, linear models were fitted, and test statistics were adjusted *via* the empirical Bayes method, with a statistical significance threshold set at *p* < 0.05 for the final identification of DEGs.

### 2.3 Protein-protein interaction (PPI) network analysis

We constructed a protein-protein interaction (PPI) network using the STRING database and visualized it in Cytoscape. Gene importance scores were computed within this network via the cytoHubba plugin, applying four algorithms: Maximum Clique Centrality (MCC), Maximum Neighborhood Component (MNC), Degree Centrality (Degree), and Edge Percolated Component (EPC). Candidate genes were identified by selecting the top 60 genes from each algorithm and determining their intersection.

### 2.4 Functional enrichment analysis

We conducted functional enrichment analysis on the differentially expressed genes using the clusterProfiler package in R. This analysis comprised Gene Ontology (GO) annotation, Kyoto Encyclopedia of Genes and Genomes (KEGG) pathway enrichment, and Gene Set Enrichment Analysis (GSEA). GO terms were examined across three categories: biological process (BP), cellular component (CC), and molecular function (MF). Enrichment results with a *p-*value <0.05 were considered statistically significant.

### 2.5 Machine learning and disease signature analysis

To explore potential core genes associated with AUD, a sequential machine learning approach was employed. First, key variables were selected using the random forest algorithm via the randomForest R package, retaining the top 15 features. These features were further refined by least absolute shrinkage and selection operator (LASSO) regression implemented with the “glmnet” package. The discriminatory performance of the resulting gene signatures and their corresponding models was assessed using receiver operating characteristic (ROC) curve analysis.

### 2.6 Immune infiltration analysis and Mendelian Randomization analysis

Computational estimation of the relative proportions of 22 immune cell subtypes in AUD and control samples was performed using CIBERSORT, a gene expression-based deconvolution algorithm. Immune cell types not detected in any sample were excluded from subsequent analyses. Associations between key genes and immune cell subtypes were evaluated using Pearson correlation analysis.

To explore potential statistical associations between key immune cell traits and AUD, Mendelian randomization (MR) analysis was performed using genetic variant data from the IEU OpenGWAS database (Table 2). Single nucleotide polymorphisms (SNPs) associated with the expression levels of key immune cell traits were selected as instrumental variables (IVs) based on the following criteria: minor allele frequency >0.05, Hardy–Weinberg equilibrium *p* < 1 × 10^−5^, and a linkage disequilibrium threshold of *r* ^2^ < 0.001 within a 10000-kb window. Where fewer than three IVs were obtained under these parameters, the significance threshold was relaxed to *p* < 1 × 10^−3^. The strength of all IVs was evaluated using the F-statistic, and an F-statistic greater than 10 was considered indicative of a strong instrument, effectively mitigating weak instrument bias.

GWAS summary statistics for both exposure and outcome traits were extracted separately from the IEU OpenGWAS database. Data harmonization was performed to ensure consistent effect allele directions for SNPs across exposure and outcome datasets. The harmonized data were further filtered to retain SNPs present in both datasets with consistent allele annotations, forming the final valid IV set for MR analysis. Multiple complementary models were used in the two-sample MR analysis to comprehensively evaluate statistical associations, including the inverse-variance weighted (IVW) method, weighted median method, MR-Egger regression, simple mode method, and weighted mode method. Associations were quantified using odds ratios (ORs) and 95% confidence intervals (CIs), and statistical significance was set at *p* < 0.05. To evaluate the reliability and robustness of the MR results, comprehensive sensitivity analyses were conducted: heterogeneity was evaluated using Cochran’s Q test under both IVW and MR-Egger frameworks, with a Q test *p* < 0.05 indicating significant heterogeneity; horizontal pleiotropy was detected via the MR-Egger regression intercept test, with an intercept *p* < 0.05 suggesting the presence of significant horizontal pleiotropy.

### 2.7 Construction of key gene regulatory network

The co-expression network was visualized with the “igraph” package in R, in which nodes correspond to key genes and edges denote significant co-expression relationships. The size of each node was scaled according to its connectivity degree within the network.

To examine potential relationships among key genes from the co-expression network, MR analysis was conducted using the methodology detailed in Section 2.6.

### 2.8 Animals

Male Long-Evans rats (initial weight: 180–220 g) were purchased from the Laboratory Animal Center of Lanzhou University. This study was approved by the Animal Ethics Committee of the School of Basic Medical Sciences, Lanzhou University (Approval No.: Lzujcyxy20240203). All rats were randomly assigned to the Naïve group, intermittent chronic ethanol drinking group (IA2BC) and chronic ethanol drinking group (2BC), with 10 animals in each group. During acclimation, rats were group-housed (3–4 per cage), followed by single housing once the alcohol drinking model was established. Animals were maintained on a 12 h/12 h light-dark cycle with the dark phase spanning 8:30 a.m. to 8:30 p.m. at a temperature of 22 °C ± 2 °C. Food was provided *ad libitum* for the whole duration of the study. Weekly body weight measurements were performed, and all experiments took place within the dark phase (9:00 a.m.–8:00 p.m.).

### 2.9 Intermittent access to 20% ethanol two-bottle free choice drinking (IA2BC) procedure

We trained rats to drink ethanol using the intermittent access two-bottle free choice (IA2BC) procedure, as described in previous studies (Li et al., 2023; Ren et al., 2023). The procedure involved giving the rats 24-h concurrent access to one bottle of 20% ethanol (EtOH, v/v) mixed with tap water and one bottle of plain water, starting at 9:00 a.m. on Mondays. After 24 h, the ethanol bottle was replaced with a second water bottle, and this pattern was repeated on Wednesdays and Fridays. On all other days, the rats had unlimited access to two water bottles. The placement of the ethanol bottle was alternated between each drinking session to control for any side preferences. Fluid consumption was measured by weighing the bottles before and after each 24-h access period for all rats. Ethanol intake was calculated as grams per kilogram of body weight. To account for fluid loss, weekly “drip” averages (measured in empty cages) were subtracted from individual intake values. Spillage remained below 1.0 mL (or less than 2.5% of total fluid intake) per 24-h period.

### 2.10 Behavioral procedure

After a minimum of 8 weeks of ethanol intake via the IA2BC paradigm, rats exhibited stable ethanol intake and ethanol preference (Supplementary Figure 1). The open field test (OFT) and elevated plus maze (EPM) were subsequently used to assess anxiety-like behaviors in two groups: rats subjected to 48-h alcohol withdrawal following IA2BC paradigm (Post-EtOH) and Naïve controls. All behavioral tests took place in the morning under controlled conditions (22 °C ± 1 °C, low noise, and uniform illumination) to minimize external disturbances. The OFT was conducted on Monday of week 9. Rats then continued ethanol consumption under the IA2BC paradigm on Wednesday and Friday of the same week. At the end of week 9, rats underwent another 48-h alcohol withdrawal. The EPM was subsequently performed on Monday of week 10, and ethanol access was restored on Wednesday and Friday of week 10. For detailed experimental procedures, please refer to the scheme and Supplementary Materials and Methods.

### 2.11 RNA isolation and RT-qPCR analysis

To explore the changes in gene expression in the central amygdala (CeA) of rats following 48 h withdrawal from chronic alcohol consumption, brain tissues were harvested on Monday in the subsequent week after the EPM test. The detailed experimental timeline is shown in the schematic diagram (Figure 4A). Rats were deeply anesthetized with sodium pentobarbital (50 mg/kg, i.p.) and transcardially perfused with ice-cold saline. The brains were then transferred to artificial cerebrospinal fluid (aCSF) saturated with carbogen (95% O_2_/5% CO_2_), containing the following components (in mM): 126 NaCl, 2.5 KCl, 1.25 NaH_2_PO_4_, 1 MgCl_2_, 2 CaCl_2_, 25 NaHCO_3_, 0.3 L-ascorbate, and 11 glucose. Coronal sections (230 μm thick) were prepared using a vibratome, and CeA-containing tissue blocks were microdissected from 3-4 sections and stored at −80 °C for subsequent analysis. Primer sequences are provided in detail in the supplementary files.

### 2.12 Correlation analysis between gene expression levels and behavioral indicators

The relative expression levels of key genes in the central amygdala (CeA)—previously identified through differential expression analysis and machine learning—were measured via RT-qPCR and served as the dependent variable. These were correlated with core behavioral metrics from the open field test (OFT) and elevated plus maze test (EPM), which were treated as independent variables. The Shapiro-Wilk normality test was first applied to assess the distribution of both gene expression data and behavioral metrics. Pearson correlation analysis was conducted for pairs of variables that conformed to a normal distribution, while Spearman rank correlation analysis was used for variables that deviated from normality. And Scatter plots with linear regression lines and 95% confidence intervals were generated to visually illustrate the association patterns between gene expression levels and each behavioral indicator. All plots were annotated with the correlation method used, the correlation coefficient (*r*), and the corresponding significance level (*p*).

### 2.13 Statistical analysis

Statistical analyses were performed using R software (version 4.2.2) and GraphPad Prism (version 9.0; GraphPad Software Inc., San Diego, CA, USA). Comparisons between two groups were conducted using unpaired Student’s t-test, while one-way analysis of variance (ANOVA) was applied for multi-group comparisons. A *p*-value <0.05 was considered statistically significant.

## 3 Results

### 3.1 Differentially expressed genes (DEGs) in the CeA with AUD

Following normalization of the GSE31708 dataset (Figure 1B), DEGs between the AUD and healthy control groups were identified using the limma package. In the CeA, a total of 251 DEGs (*p* < 0.05) were identified (Supplementary Table 1). The associated volcano plot (Figure 1C) showed 113 upregulated and 138 downregulated genes, while a heatmap of the top 50 relatively pronounced altered DEGs is presented in Figure 1D.

### 3.2 Identification of hub genes within the PPI network constructed from CeA DEGs

**FIGURE 2:**
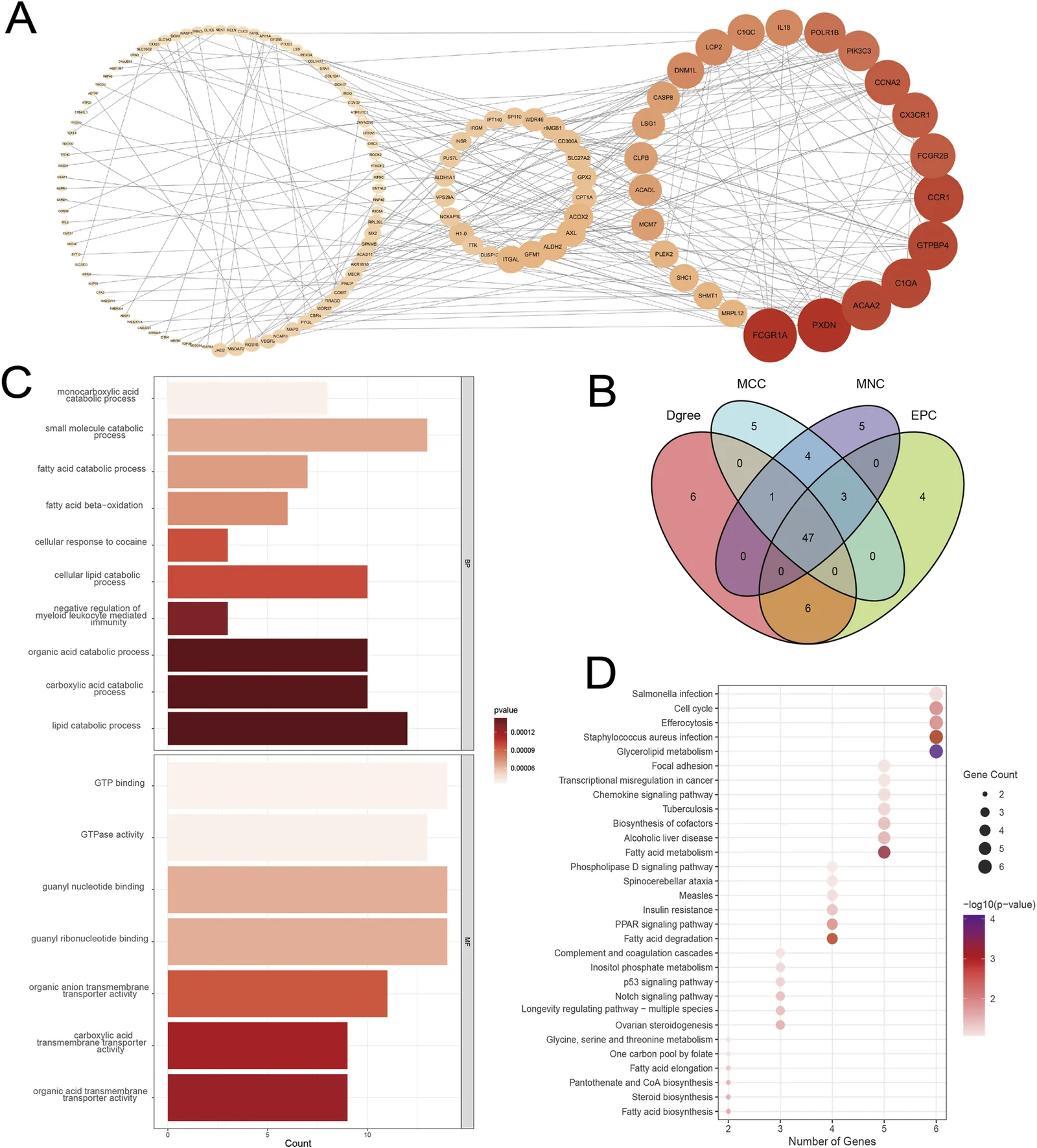
Protein-protein interaction (PPI) network and hub gene analysis. **(A)** The module with the highest weight in the PPI network constructed using the STRING database, where gene weight is positively correlated with the diameter and color intensity of the circles (132 nodes and 229 interaction edges). **(B)** Venn diagram showing the identification of 47 common hub genes by intersecting the top 60 genes weighted by four algorithms (MCC, MNC, Degree, EPC) in the cytoHubba plugin. **(C,D)** Gene Ontology (GO) and Kyoto Encyclopedia of Genes and Genomes (KEGG) enrichment analyses of hub genes (*p* < 0.05).

To further explore AUD-related candidate genes in the CeA, PPI network analysis of the differentially expressed genes was performed using the STRING database. This analysis produced a network of 132 nodes (candidate genes) with 229 interaction edges (protein–protein interactions) (Figure 2A). Employing four algorithms within the cytoHubba plugin, we identified 47 candidate genes that were consistent across all methods, following a screen of the top 60 candidates from each algorithm. (Figure 2B). Functional enrichment analysis of the candidate genes indicated they were significantly enriched in specific terms, particularly the lipid catabolic process (Biological Process,BP,*p* = 0.0001) and GTP binding (Molecular Function,MF,*p* < 0.0001), as visualized in Figure 2C. Kyoto Encyclopedia of Genes and Genomes (KEGG) pathway analysis suggested significant enrichment in pathways related to Glycerolipid metabolism (Gene count = 6,*p* < 0.0001) (Figure 2D).

### 3.3 Validation of hub genes within the CeA

**FIGURE 3:**
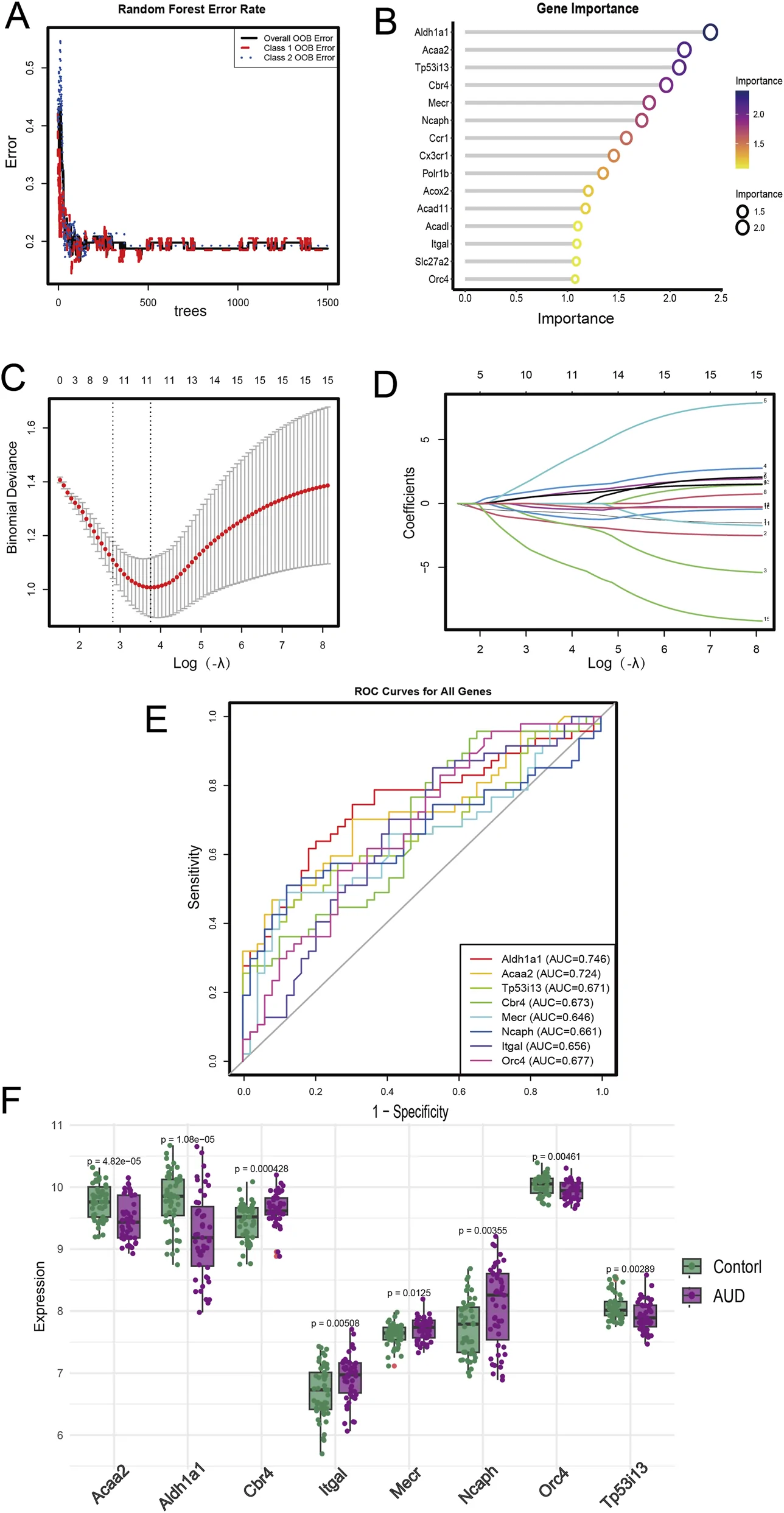
Identification of key genes associated with AUD. **(A,B)** Top 15 genes identified from hub genes using the random forest (RF) algorithm based on importance ranking. **(C,D)** Eight key genes with Non-zero coefficients identified from the 15 genes *via* LASSO regression (λ = 3.31). **(E)** Receiver operating characteristic (ROC) curves of the eight key genes, all with AUC > 0.65, indicating good diagnostic efficacy for AUD. **(F)** Boxplots of the eight key genes based on unpaired t-tests.

Machine learning algorithms were applied to further screen and screen candidate DEGs in the CeA. The random forest (RF) algorithm identified the top 15 genes based on importance ranking (Figures 3A,B), and LASSO regression (λ = 3.31) selected eight exploratory molecular indicators--*Acaa2, Aldh1a1, Cbr4, Itgal, Mecr, Ncaph, Tp53i13, Orc4* (Figures 3C,D). These candidates were then examined by using ROC curve analysis, with all exhibiting AUC values >0.65, suggesting moderate discriminatory potential for AUD (Figure 3E).

**FIGURE 4:**
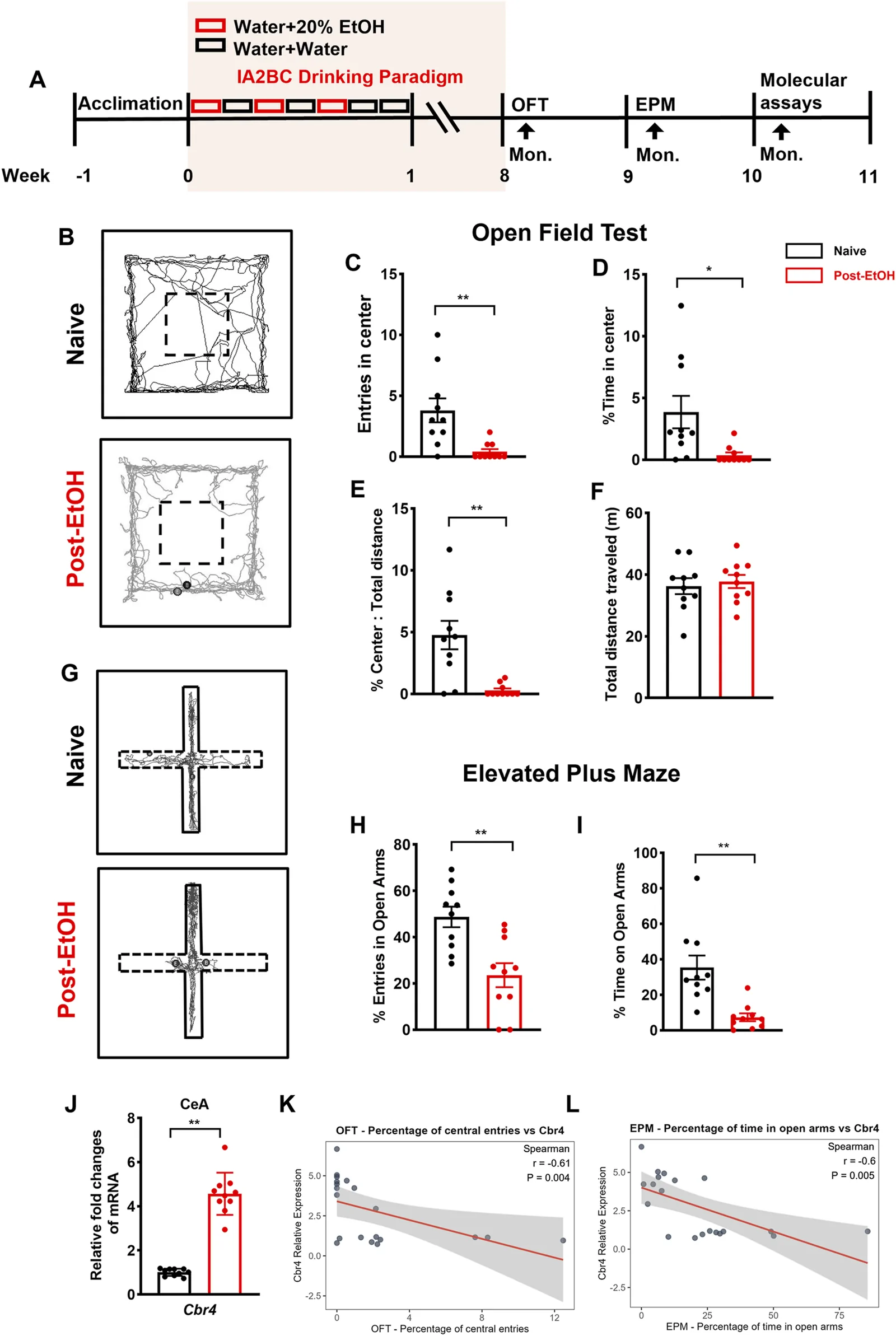
Behavioral tests, RT-qPCR validation of key genes, and correlation analysis in the CeA. **(A)** The schematic diagram shows the experimental timeline. gradually escalating from the 1st to the 24th session and reaching stable levels (*p < 0.05, **p < 0.01 vs. 1st session, one-way RM ANOVA followed by Bonferroni post-hoc test). **(B)** Representative trajectory map of the Open Field Test (OFT) in Naïve and Post-EtOH rats. **(C–F)** Comparison of four key indicators of the OFT between the two groups: Post-EtOH rats showed elevated anxiety-like behaviors compared with Naïve rats (*p < 0.05, **p < 0.01 vs. Naïve group, Unpaired t-test). **(G)** Representative trajectory map of the Elevated Plus Maze (EPM) in Naïve and Post-EtOH rats. **(H–I)** Comparison of two key indicators of the EPM between the two groups: Post-EtOH rats exhibited increased anxiety-like behaviors compared with Naïve rats (*p < 0.05, **p < 0.01 vs. Naïve group, Unpaired t-test). **(J)** RT-qPCR validation of the key candidate gene in the CeA: *Cbr4* showed significant expression differences between the Naïve and Post-EtOH groups. (*p* < 0.05, Unpaired t-test). **(K–L)** Pearson correlation analysis between the key gene *Cbr4* and core behavioral indicators: **(K)** Correlation between *Cbr4* and the percent of time in center (OFT); **(L)** Correlation between Cbr4 and the percent of time in the open arms (EPM), both showing a significant negative correlation (*p* < 0.05).

We trained male Long-Evans rats (*n* = 10) to consume 20% ethanol under the intermittent access to 2-bottle choice (IA2BC) paradigm (Figure 4A). A Naïve group (*n* = 10) received only water under identical housing and experimental conditions. Over the 8-week training period, alcohol-drinking animals exhibited a gradual and significant escalation in ethanol intake, increasing from 1.01 ± 0.11 g/kg/24 h in the first session to 7.32 ± 1.03 g/kg/24 h in the 24th session, after which intake stabilized at high levels (Supplementary Figure 1). Following the establishment of stable ethanol consumption, a battery of behavioral tests including the open field test (OFT) and elevated plus maze (EPM) was conducted at 48 h into alcohol withdrawal (Post-EtOH group) (Figures 4A–I). Behavioral analysis revealed that Post-EtOH rats displayed elevated anxiety-like behaviors compared to Naïve controls (Figures 4B–I).

Based on these behavioral phenotypes, we next measured the mRNA expression levels of the eight candidate genes previously identified through machine learning in the CeA of Naïve and Post-EtOH rats. All eight genes were found to be expressed in the CeA (Supplementary Figure 2). Among them, only *Cbr4* and *Itgal* exhibited statistically significant expression differences between the two groups (*p* < 0.05) (Figure 4J; Supplementary Figure 2). Notably, *Cbr4* showed consistent and significant upregulation across both gene microarray and RT-qPCR analyses, whereas *Itgal* exhibited a divergent expression pattern between the two detection methods (Figure 3F; Supplementary Figure 2). Consistent with its differential expression, receiver operating characteristic (ROC) analysis suggested that *Cbr4* achieved an area under the curve (AUC) of 0.673 (Figure 3E), suggesting its potential discriminatory capacity for AUD. To further explore the functional relevance of *Cbr4* upregulation, we examined the correlation between *Cbr4* expression levels in the CeA and anxiety-related behavioral metrics. As shown in Figures 4K,L, *Cbr4* expression was significantly negatively correlated with two core anxiety indicators: the percentage of time spent in the central zone of the OFT (*r* = −0.613, Spearman *p* = 0.004) (Figure 4K) and the percentage of time spent in the open arms of the EPM (*r* = −0.605, Spearman *p* = 0.005) (Figure 4L).

To clarify whether the altered expression of *Cbr4* in the CeA arises from alcohol withdrawal or the direct effects of chronic alcohol exposure, we established an additional Non-abstinent group that received continuous 20% ethanol administration for 8 weeks (2BC) without any withdrawal period (Supplementary Figure 7A). One-way ANOVA analysis revealed that *Cbr4* expression was increased in the Post-EtOH group relative to both Naïve and Non-abstinent groups (*p* < 0.01). Tukey’s Honestly Significant Difference (Tukey’s HSD) Post-hoc test further confirmed no significant difference in *Cbr4* expression between Naïve and Non-abstinent rats (Supplementary Figure 7B). Collectively, these findings suggest that the upregulation of *Cbr4* in the CeA may be associated with alcohol withdrawal, rather than chronic alcohol exposure perse.

Furthermore, we investigated the brain region specificity of *Cbr4* expression changes. No significant differences in *Cbr4* expression were observed in the medial prefrontal cortex (mPFC), hippocampus, or ventral tegmental area (VTA) between Naïve and Post-EtOH rats (Supplementary Figure 6), indicating that *Cbr4* upregulation following chronic alcohol drinking is specific to the CeA. Taken together, these findings preliminarily suggest that *Cbr4* may be involved in processes associated with alcohol use disorder.

### 3.4 Integrative GSEA and molecular network analysis of key genes in the CeA

**FIGURE 5:**
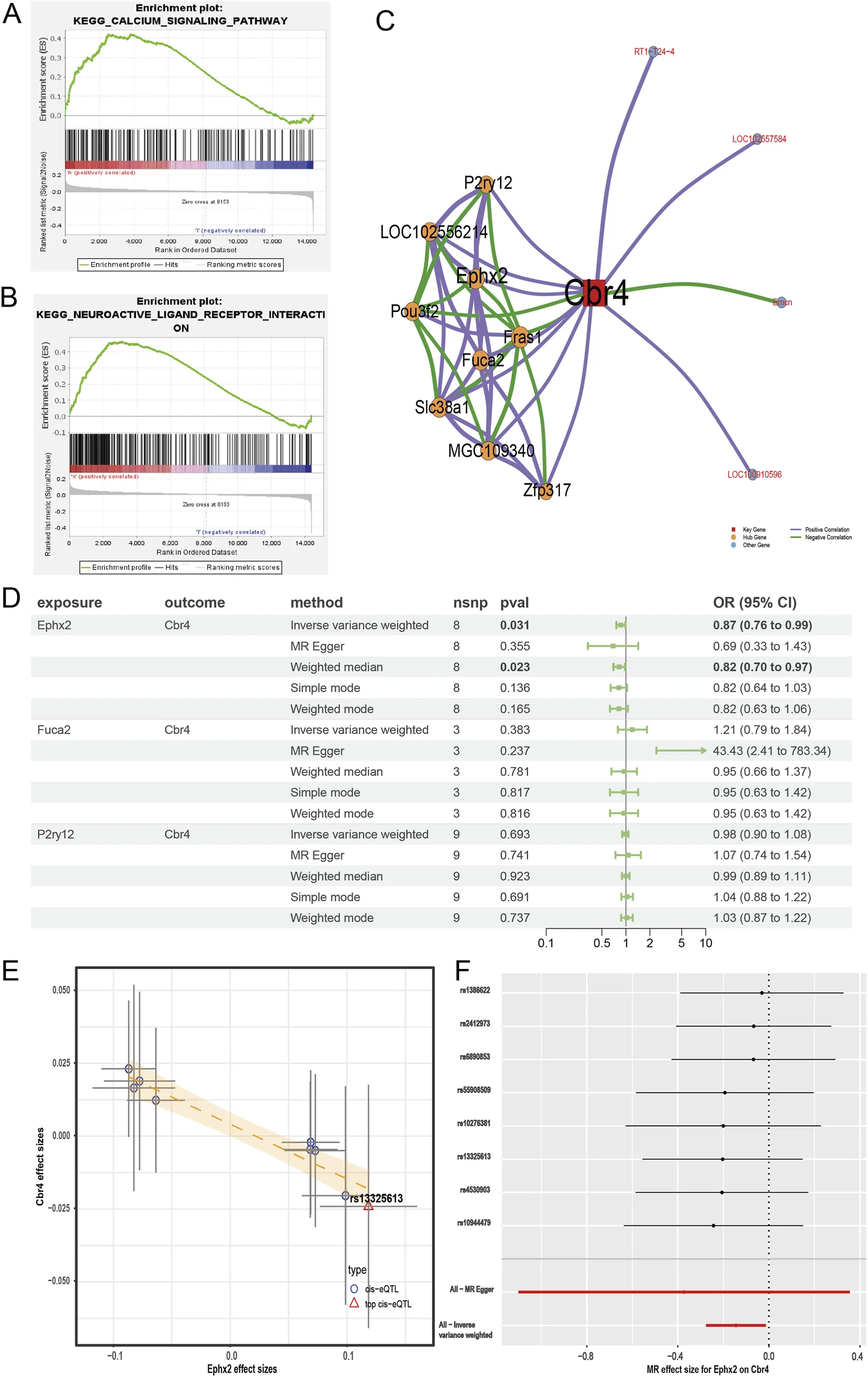
Functional enrichment, co-expression network, and MR analysis of *Cbr4*. **(A,B)** Key pathways from Gene Set Enrichment Analysis (GSEA) of *Cbr4*: **(A)** calcium ion signaling pathway (NES = 1.50, *p* = 0.027); **(B)** neuroactive ligand-receptor interaction pathway (NES = 1.45,*p* = 0.037). **(C)** Co-expression network of *Cbr4* in the GSE31708 dataset (threshold |r| > 0.6), comprising 14 nodes and 32 edges, with *Cbr4* as the central hub. **(D)** Expression quantitative trait locus (eQTL)-based Mendelian randomization (MR) analysis between genes in the co-expression network and *Cbr4*. **(E)** Scatter plot of eQTL-MR analysis between *Ephx2* and *Cbr4.* **(F)** Forest plot of single nucleotide polymorphisms (SNPs) from eQTL-MR analysis between *Ephx2* and *Cbr4*. The plot displays the number of instrumental variables, exposure-outcome factors, and effect size statistics. The inverse variance weighted (IVW) model showed a positive causal effect (OR = 1.32, 95% CI = 1.15–1.51, *p* = 2.3 × 10^−4^).

To explore pathways associated with *Cbr4* expression in AUD progression, we performed Gene Set Enrichment Analysis (GSEA) (Supplementary Tables 2,3). The analysis identified significant upregulation of the calcium signaling pathway (NES = 1.50, *p* = 0.027) (Figure 5A) and the neuroactive ligand-receptor interaction pathway (*p* = 0.037) (Figure 5B), suggesting a potential association between *Cbr4* and neuronal excitability and synaptic communication. And a *Cbr4*-centric co-expression subnetwork was constructed from the key gene expression matrix of the GSE31708 dataset, applying a threshold of *|r|* > 0.6. This subnetwork comprised 14 nodes and 32 edges, suggesting notable co-expression relationships. Within this subnetwork, *Cbr4* shows notable co-expression with *Ephx2*, *Slc38a1*, and *Pou3f2*, suggesting potential associations between *Cbr4* and these genes (Figure 5C).

Focusing on the core node *Cbr4* and its directly connected pairs, we conducted two-sample Mendelian Randomization (MR) analysis to interrogate statistical evidence for potential associations within the subnetwork (Figure 5D). From AUD-relevant GWAS data (IEU database), we selected instrumental variables (IVs) meeting standard criteria. Employing the Inverse Variance Weighted (IVW) model as primary analysis, our MR analysis showed a statistical association between genetically predicted *Ephx2* and *Cbr4* expression. Specifically, under the IVW model, higher *Ephx2* levels were associated with increased *Cbr4* expression (OR = 1.32, 95% CI = 1.15–1.51, *p* = 2.3 × 10^−4^). Results from the weighted median model aligned with the primary analysis (OR = 1.28, 95% CI = 1.07–1.53, *p* = 0.006). Sensitivity analyses ruled out significant horizontal pleiotropy (*p* = 0.41) and heterogeneity (*p* = 0.28), thereby upholding the reliability of the statistical inference. These findings provide statistical evidence for a potential functional link: *Cbr4* and *Ephx2* may be involved in lipid metabolism dysregulation and may play a role in the pathological progression of AUD mediated by the CeA. Notably, the identified association between these two genes offers a potential entry point for dissecting the broader *Cbr4*-centered regulatory network in AUD pathogenesis.

### 3.5 Immune cell infiltration analysis in the CeA

**FIGURE 6:**
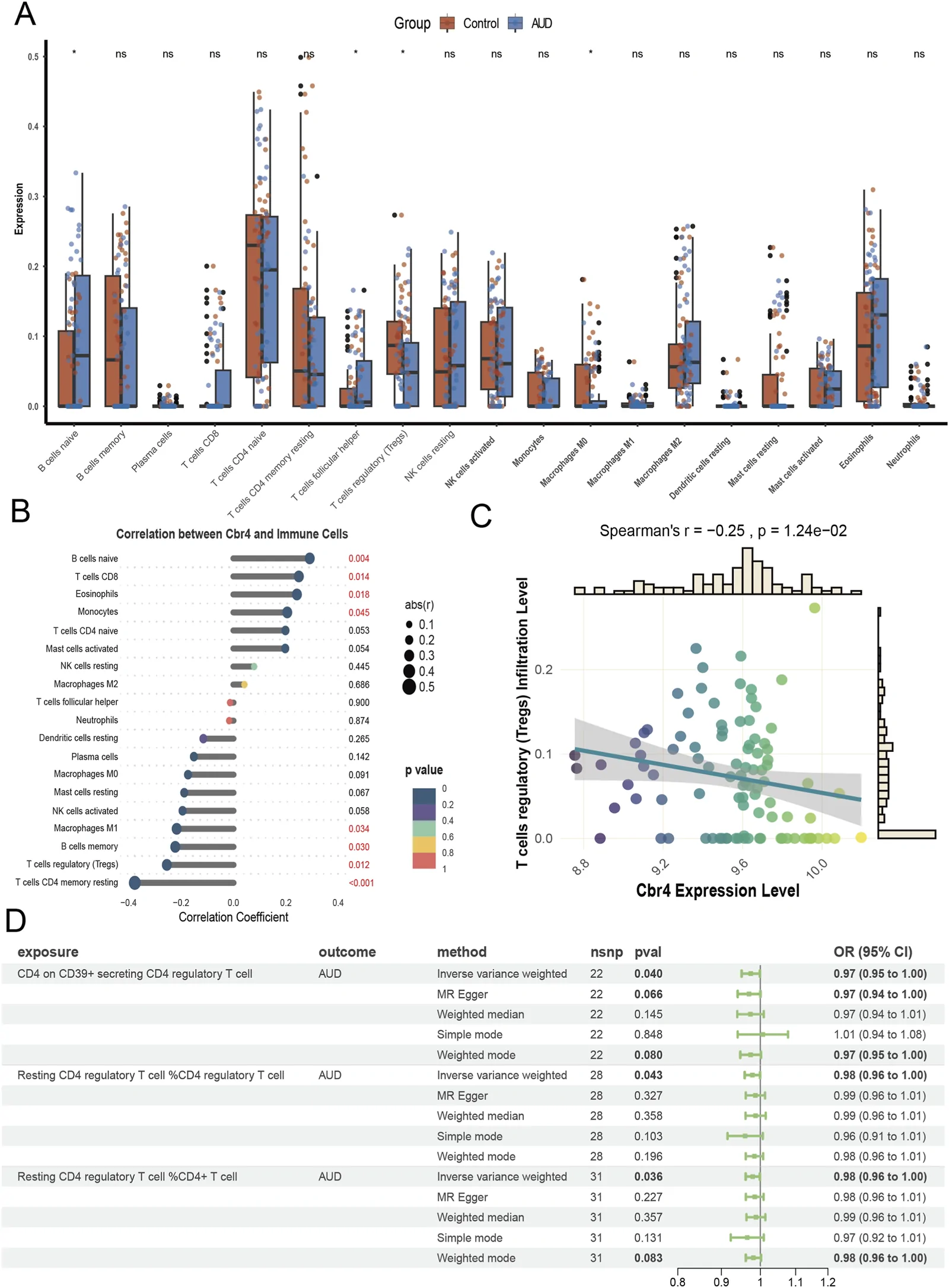
Immune cell-related analysis and Mendelian randomization (MR) study. **(A)** Boxplots of differential analysis of immune cells in the CeA: the AUD group showed increased proportions of Naïve B cells and T follicular helper (Tfh) cells, and a decreased proportion of regulatory T cells (Tregs) compared with the control group (*p < 0.05, **p < 0.01 vs. Control group, Unpaired t-test). **(B)** Lollipop plot showing the correlation between *Cbr4* and each immune cell type: *Cbr4* was inversely associated with Tregs (r = −0.25) and positively linked to Naïve B cells (r = 0.23). **(C)** Scatter plot of the correlation between *Cbr4* and Tregs (r = −0.25, *p* = 0.012, Pearson correlation analysis). **(D)** Mendelian randomization (MR) analysis between multiple Treg subsets and AUD: all Treg subtypes exerted a protective effect against AUD (IVW method: OR = 0.9744–0.9797, *p* = 0.036–0.043).

Notably, GSEA enrichment analysis of *Cbr4* revealed that the IgA production pathway was significantly enriched (*p* = 0.006) (Supplementary Table 2), suggesting that *Cbr4* may be associated with immune dysregulation in the pathogenesis of AUD. To investigate immune alterations in the amygdala of AUD subjects and the associations between *Cbr4* and immune cell subtypes, we conducted immune infiltration analysis. The CIBERSORT algorithm was utilized to quantify the immune cell composition within the CeA of both AUD and control subjects (Supplementary Table 8). Computational prediction suggested increased Naïve B and T follicular helper (Tfh) cells, alongside decreased regulatory T cells (Tregs), relative to healthy controls (Figure 6A). The key gene *Cbr4* was inversely associated with Tregs levels (r = −0.25, *p* = 0.012) but was positively linked to the proportion of Naïve B cells (r = 0.23, *p* = 0.004) (Figures 6B,C).

Furthermore, focusing on the two immune cell subtypes with the most prominent differences identified by CIBERSORT—Naïve B cells and regulatory T cells (Tregs)—we performed two-sample Mendelian randomization (MR) analysis to explore potential associations with AUD. Mendelian randomization analysis revealed significant statistical associations between multiple Tregs subtypes and AUD, all of which showed a protective direction (IVW method: OR = 0.9744–0.9797, *p* = 0.036–0.043). By contrast, no such association was found for Naïve B cells (IVW method: OR = 0.9896–1.0052, *p* = 0.330–0.707) (Supplementary Figure 5). Tregs depletion, as suggested by the concordance between MR and transcriptomic data (Figure 6D), may be involved in a pathogenic cascade.

## 4 Discussion

The central amygdala directly mediates compulsive alcohol-seeking and relapse behaviors in patients with AUD by modulating the negative reinforcement effects and aversive responses induced by alcohol withdrawal (Roberto et al., 2021). Despite considerable advances in relevant research, effective CeA-targeted potential molecular indicators remain to be identified. Emerging evidence indicates that the dysregulated bidirectional control of the metabolic-immune microenvironment in the central nervous system may be one of the pathological mechanisms associated with AUD (Ghare et al., 2011; Mews et al., 2019; Jeon and Carr, 2020; Crotty et al., 2023; Paterson et al., 2023; Bazargan-Hejazi et al., 2024; Togre et al., 2025; Vella et al., 2025). Using the GEO database, this study applied multi-dimensional bioinformatics analyses to explore CeA-specific candidate genes in AUD subjects. Through in-depth analysis of the GSE31708 dataset, a PPI network of CeA differentially expressed genes was constructed, yielding 47 candidate key genes. Subsequent feature selection via LASSO regression and random forest cross-validation suggested nine candidate genes with potential relevance to AUD. Chronic alcohol exposure animal model validation suggested that CeA *Cbr4* expression was significantly altered and correlated with alcohol withdrawal-induced anxiety-like behaviors. Exploratory analysis via Mendelian randomization suggested that *Ephx2* may be associated with CeA *Cbr4* expression, while immune infiltration analysis suggested that aberrant *Cbr4* expression was closely linked to Treg cells function-related changes. These findings provide insights into potential CeA-specific molecular correlates of AUD and offer preliminary clues for further investigation.

The *Cbr4* (Carbonyl Reductase 4) gene maps to the 1p36.33 locus on human chromosome 1, and its encoded protein is a member of the short-chain dehydrogenase/reductase (SDR) superfamily (Endo et al., 2008; Malátková et al., 2010). This enzyme possesses dual catalytic activities: it mediates the reduction of a broad spectrum of carbonyl compounds and exhibits robust quinone reductase activity (Endo et al., 2008). During the reductive metabolism of quinones, the reaction yields superoxide anions, a finding that suggests *Cbr4* may be associated with alcohol-induced oxidative stress and subsequent exacerbation of alcohol-associated toxic insults to neural tissues and other organ systems (Endo et al., 2008).Furthermore, GSEA results suggest a potential association between *Cbr4* and intracellular calcium overload in the context of AUD.

Following functional characterization of *Cbr4*, we constructed a Pearson correlation-based co-expression network, and Mendelian randomization analysis suggested a significant correlation and genetic association between *Cbr4* and *Ephx2*. *Ephx2* encodes soluble epoxide hydrolase (sEH), which catalyzes the conversion of arachidonic acid-derived anti-inflammatory/neuroprotective epoxyeicosatrienoic acids (EETs) into less bioactive dihydroxyeicosatrienoic acids (DHETs) (Liu et al., 2021; Edin et al., 2023; Wm et al., 2023). EETs act as an endogenous “repair system” counteracting central lipid homeostasis dysregulation from chronic alcohol exposure, via sEH inhibition (Iyer et al., 2022; Smith et al., 2022). Previous studies have demonstrated that soluble epoxide hydrolase (sEH) inhibitors can regulate tight junctions, potentially improve blood-brain barrier endothelial permeability, and block the entry of peripheral inflammatory cytokines and harmful substances into the central nervous system, possibly thereby conferring a protective effect against alcohol-mediated brain injury (Kuo and Lee, 2022; Li et al., 2024). It is worth noting that sEH is also expressed in neurons of the central nucleus of the amygdala (CeA) and may contribute to the regulation of anxiety-like behaviors, while the specific molecular mechanism governing this process remains highly complex (Ren et al., 2022). Notably, MR analysis suggested a statistical association between *Ephx2* and *Cbr4,* which is generally consistent with the potential mechanisms of AUD. Collectively, we hypothesize that *Cbr4* and *Ephx2* may be involved in processes associated with the pathogenesis of alcohol use disorder by jointly modulating lipid metabolism dysregulation.

As a core brain region integrating emotion and reward processing, the CeA nucleus exhibits a highly sensitive neuroimmune microenvironment to alcohol exposure, and aberrant immune signaling acts as a key mechanism mediating CeA dysfunction and progression of AUD (Melkumyan et al., 2025). Immune infiltration analysis in the present study using computational prediction suggested a reduced proportion of regulatory T cells (Tregs) in the CeA of AUD subjects. Further analysis via two-sample MR indicated that multiple Tregs-associated immunophenotypes may be potentially associated with reduced AUD risk, whereas lower Treg levels may correlate with a multi-tiered pathogenic cascade, potentially accelerating disease progression. Moreover, accumulating evidence has demonstrated that impaired Tregs function attenuates its inhibitory effect on neuroinflammation within the CeA, and aberrant secretion of immunoregulatory factors such as *IL-10* and *TGF-β* further disrupts CeA immune homeostasis, potentially exacerbating alcohol-induced brain injury (Knapp et al., 2011; Marshall et al., 2017; Patel et al., 2021). Correlation analysis between the hub gene *Cbr4* and immune cells further suggested that it may be associated with immune homeostasis imbalance in the CeA through potential effects on Tregs function, suggesting that *Cbr4* may serve as a candidate intermediate factor linking immune dysfunction to neural damage. This finding further supports the potential role of immunological mechanisms in the pathogenesis of AUD.

From a clinical application perspective, the findings of this study hold potential practical implications. Currently, the diagnosis of AUD still relies on clinical interviews and scale assessments—traditional methods characterized by prominent subjectivity, susceptibility to social desirability bias, and a lack of objective biological indicators (Coriale et al., 2018; Witkiewitz et al., 2024). In contrast, the characteristics of *Cbr4* identified in this study provide a potential objective basis for exploring CeA-specific molecular indicators for AUD, offering clues for improving the standardization and accuracy of AUD diagnosis. Furthermore, the association between *Cbr4* and *Ephx2*, opens up new avenues for the development of personalized candidate agents, particularly suitable for AUD subpopulation with EETs metabolic dysfunction within the CeA. Meanwhile, the differential immune cell profile further suggests that modulating Tregs activity in the CeA to restore central immune homeostasis may provide a potential theoretical basis for immunomodulatory interventions for specific AUD subgroups.

This study has several important limitations that should be duly acknowledged. First, the GSE31708 dataset used in this work comprises samples from multiple selectively bred rat lines with distinct genetic backgrounds. While this design enhances the generalizability of findings across different genetic contexts, it also introduces potential confounding factors, as observed gene expression differences may reflect both alcohol preference-related traits and inherent strain-specific genetic variations. Additionally, we identified differentially expressed genes using an unadjusted *p*-value threshold of 0.05 due to relatively weak differential expression signals in this dataset. This lenient statistical threshold increases the risk of false-positive results, although we validated the expression changes of the key gene *Cbr4* in an independent animal model. Second, our study only confirmed the aberrant upregulation of *Cbr4* in the central amygdala (CeA) during alcohol withdrawal and its correlation with anxiety-like behaviors. Crucially, we did not perform CeA-specific functional manipulation of *Cbr4* (such as conditional overexpression or knockout) to verify its causal role in AUD pathogenesis. We also did not conduct direct measurements of lipid metabolites in the CeA to experimentally validate the hypothesized involvement of *Cbr4* and *Ephx2* in lipid metabolism dysregulation. Furthermore, the functional status of regulatory T cells (Tregs) in the CeA was not experimentally assessed; the observed alterations in Treg proportions represent computational predictions based on transcriptomic deconvolution rather than direct immunological measurements. Third, the exploratory nature of our bioinformatic analyses warrants explicit emphasis. Mendelian randomization (MR) analysis provides statistical evidence consistent with potential causal relationships under specific assumptions, but it does not establish direct biological causality. Similarly, CIBERSORT-based immune infiltration results are computational inferences that require independent experimental validation. Furthermore, the diagnostic performance of *Cbr4* for AUD identified in this study was moderate (AUC = 0.673), and its clinical utility needs to be further validated in large-scale, independent clinical cohorts. Finally, we observed discrepant expression patterns of *Itgal* between microarray and RT-qPCR analyses, which represents a technical limitation of this study. Such inconsistency is common in transcriptomic validation studies and can be attributed to differences in detection principles, probe/primer targeting regions, and potential alternative splicing events induced by chronic alcohol exposure.

## 5 Conclusion

Through the integration of multi-dimensional bioinformatics analyses and *in vivo* validation experiments, this study suggests that *Cbr4* may be a putative gene in the CeA that may contribute to the pathological processes of AUD. Aberrantly upregulated *Cbr4* correlates with dysregulated EETs metabolism and immune dysfunction in the CeA. These findings expand our preliminary understanding of AUD pathogenesis and provide exploratory clues for potential molecular indicators and related strategies for AUD. Further investigations are warranted to validate these findings in larger multi-center cohorts and elucidate the underlying molecular mechanisms.

## Data Availability

The original contributions presented in the study are publicly available. This data can be found in the Gene Expression Omnibus repository with the accession number GSE31708.
