# Correction: Integrative bioinformatics and in vivo validation suggest a potential role of *Cbr4* in alcohol use disorder through modulation of lipid metabolism and treg cell function in the central amygdala

**DOI:** 10.3389/fgene.2026.1975571

**Published:** 2026-09-08

**Authors:** Yao Ge, Yihan Yuan, Min Li, Zhenxia Huang, Chenli Zhang, Caiyu Yu, Jinyu Xu, Siao Zhang, Hongwei Guo, Xu Zhang, Rao Fu, Zhiheng Ren

**Affiliations:** 1 Department of Pathology, School of Basic Medical Sciences, Lanzhou University, Lanzhou, Gansu, China; 2 The First School of Clinical Medicine, Lanzhou University, Lanzhou, Gansu, China; 3 The Second Clinical Medical School, Lanzhou University, Lanzhou, Gansu, China; 4 Department of Pathology, Qingyuan Hospital, Guangzhou Medical University, Qingyuan, Guangdong, China; 5 Department of Neurology, The Second Hospital of Lanzhou University, Lanzhou, Gansu, China; 6 Department of Anatomy, School of Medicine, Shenzhen Campus of Sun Yat-sen University, Sun Yat-sen University, Shenzhen, Guangdong, China

**Keywords:** alcohol use disorder, *Cbr4*, central amygdala, lipid metabolism, mendelian randomization, regulatory T cells

Affiliation The Second Clinical Medical School, Lanzhou University, Lanzhou, Gansu, China was omitted from the paper. This affiliation has now been added for author(s) Yihan Yuan.

The original version of this article has been updated.

