# Supplementary material for: Integrative bioinformatics and in vivo validation suggest a potential role of Cbr4 in alcohol use disorder through modulation of lipid metabolism and treg cell function in the central amygdala
Source: Front Genet. 2026 Jul 1;17:1829878. doi: 10.3389/fgene.2026.1829878 (PMC13368475; doi:10.3389/fgene.2026.1829878)
Supplement: Supplementary file 2 [file DataSheet1.docx]

Supplementary Files

1. Supplementary Figures


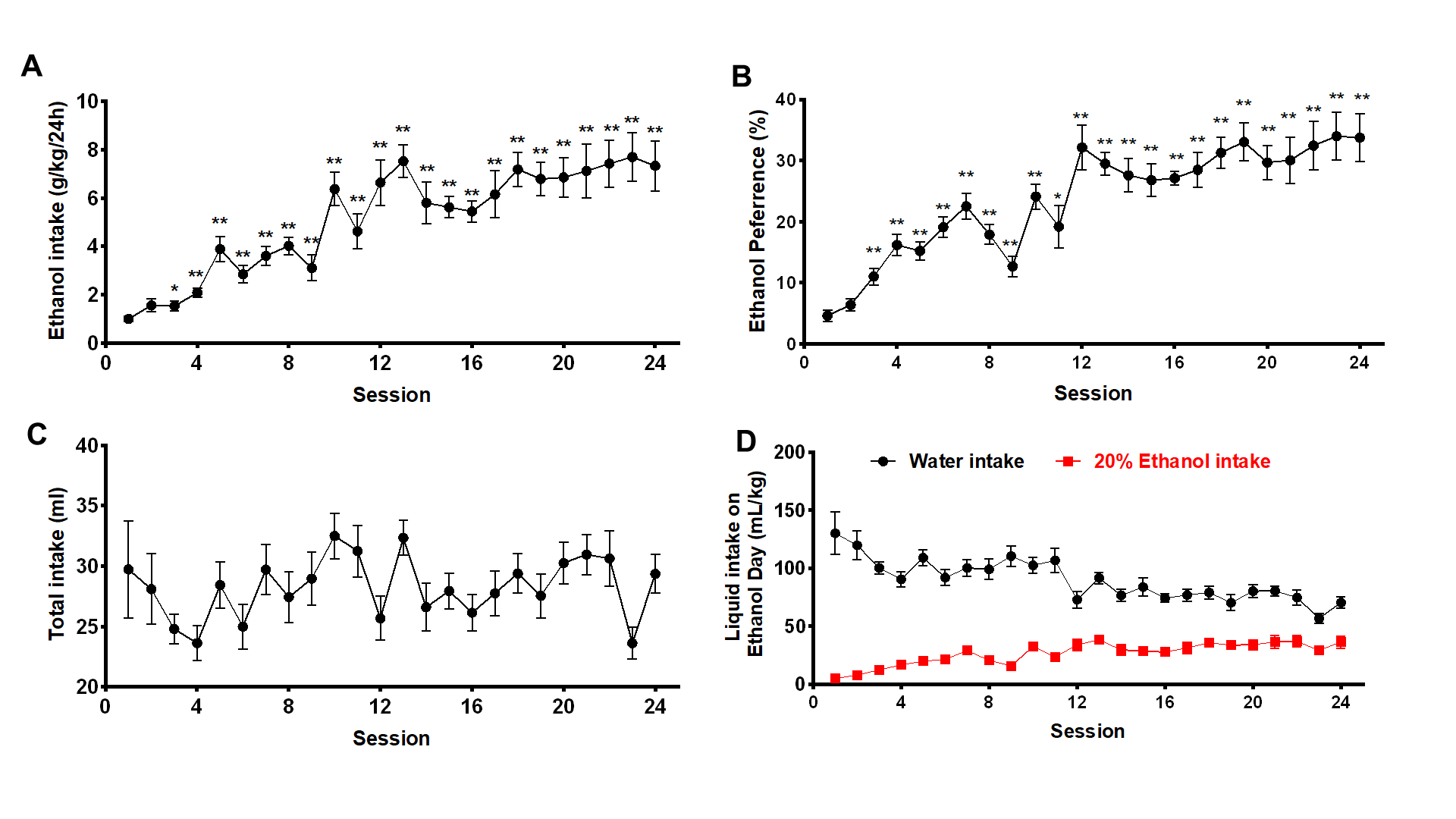


Supplementary Figure 1. A chronic alcohol consumption rat model was established using the IA2BC drinking paradigm. Rats were trained to voluntarily consume 20% (v/v) ethanol in the IA2BC paradigm for 8 weeks. The panels summarized ethanol intake (A), ethanol preference (B), total fluid intake (C), and fluid intake on ethanol day(D). **p*<0.05 and ***p*<0.01 vs. 1st session, revealed by one-way RM ANOVA followed with Bonferroni posthoc test.


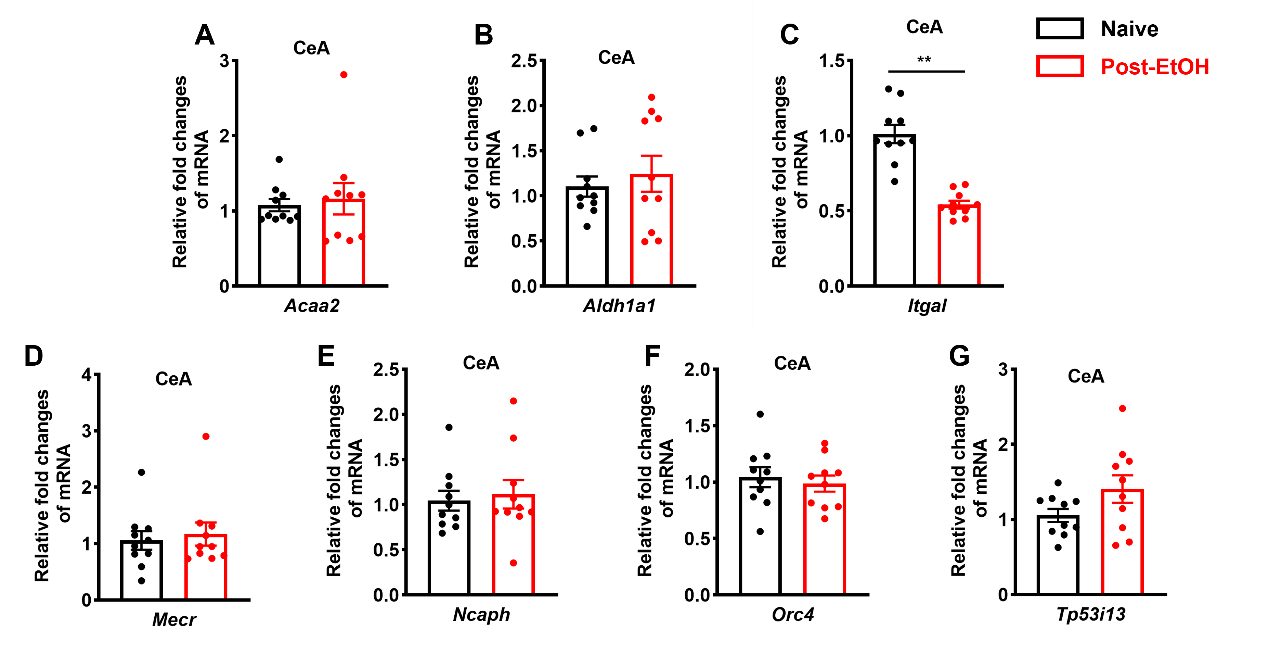


Supplementary Figure 2. RT-qPCR validation of the additional 7 genes identified via machine learning within the central amygdala (CeA). The central amygdala (CeA) was dissected from the brain tissues of Naive rats and alcohol-withdrawn rats (Post-EtOH) for RT-qPCR analysis. Panels A–G depict the relative mRNA fold changes of candidate genes in the CeA of Naive (black) and Post-EtOH (red) rats. *p* < 0.01 versus the Naive group, as determined by unpaired t-test, n=10 rats/group. All data are shown as mean ± SEM.


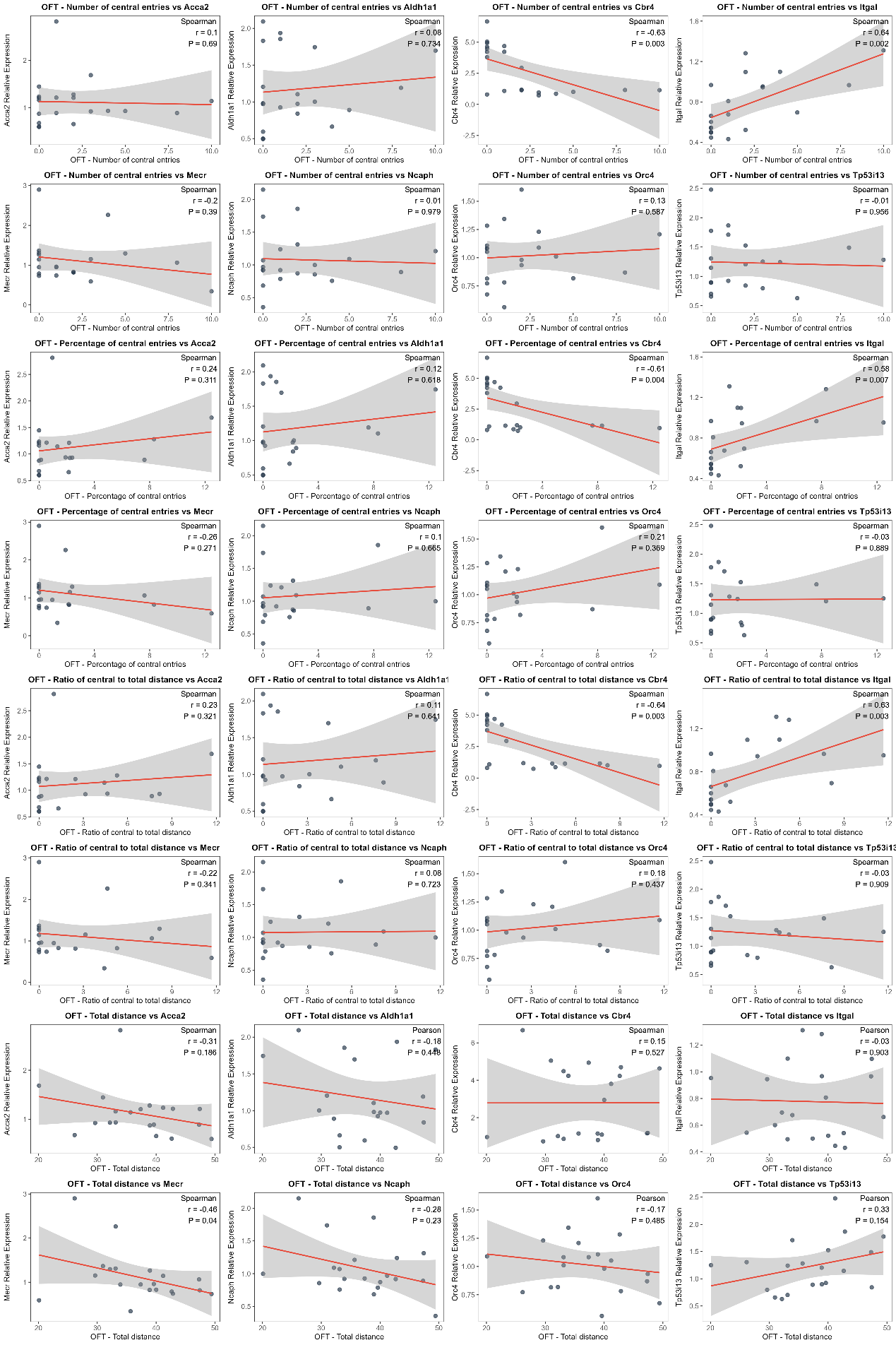


Supplementary Figure 3. Correlation analyses were conducted between four key indicators of the Open Field Test (OFT) and 8 genes screened via machine learning. The panels display correlation scatter plots for each OFT indicator with the 8 genes. Pearson/Spearman correlation coefficients (R) and corresponding *P*-values are labeled in each subplot, with significant correlations marked.


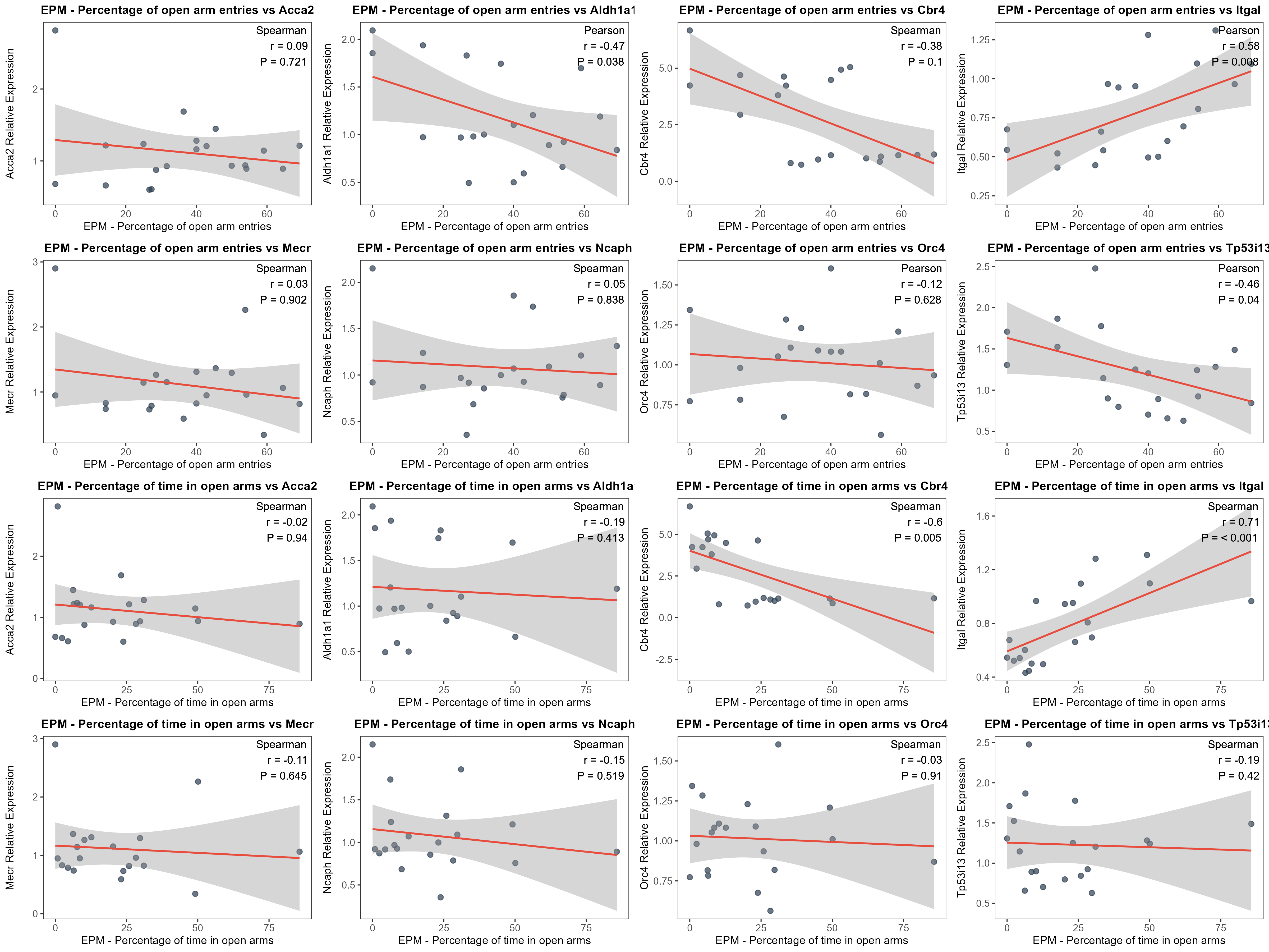


Supplementary Figure 4. Correlation analyses were conducted between two key indicators of the Elevated Plus Maze (EPM) Test and 8 genes screened via machine learning. The panels display correlation scatter plots for each EPM indicatorwith the 8 target genes. pearman/Spearman Spearman correlation coefficients (R) and corresponding *P*-values are labeled in each subplot, with significant correlations marked.


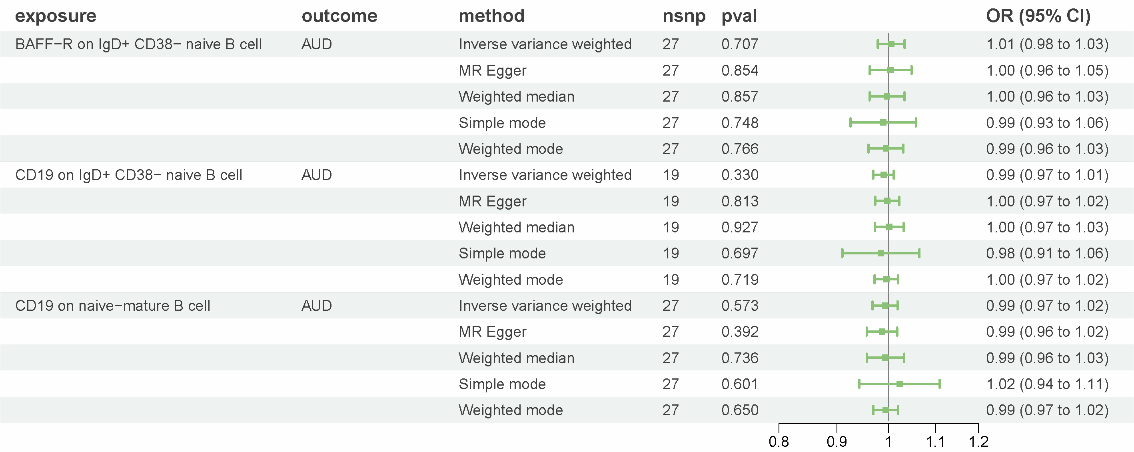


Supplementary Figure 5: Mendelian randomization forest plot of naive B cells and AUD. Two-sample MR analysis showed no significant causal link between naive B cells and AUD (IVW method: OR = 0.9896–1.0052, *p* = 0.330–0.707).


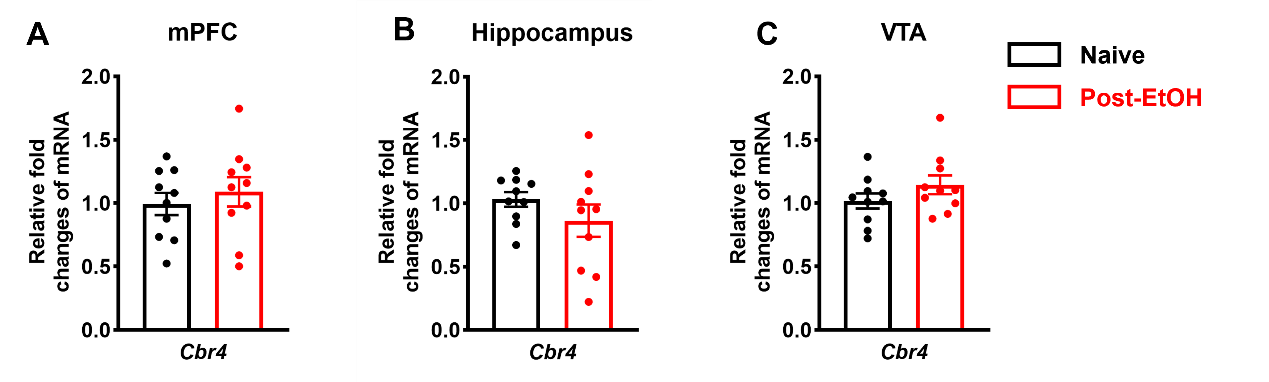


Supplementary Figure 6

Comparison of *Cbr4* expression in the mPFC, hippocampus and VTA.

*Cbr4* mRNA expression levels were measured in the mPFC, hippocampus, and VTA brain regions from Naïve and Post-EtOH rats. Unpaired t-test revealed no significant differences in *Cbr4* expression in the mPFC (A), hippocampus (B), and VTA (C) between the Naive and Post-EtOH groups, n=10 rats/group. All data are shown as mean ± SEM (During the revision period, only quantitative detection and statistical analysis of *Cbr4* expression in the mPFC, VTA and Hippocampus were performed on pre-collected tissue samples).

Our results revealed that *Cbr4* expression in the CeA was significantly upregulated in Post-EtOH rats relative to Naïve controls. To determine whether altered *Cbr4* expression is specifically associated with alcohol withdrawal, we quantified the relative mRNA levels of *Cbr4* in the CeA of rats under the two-bottle choice (2BC) paradigm without abstinence (Supplementary Figure 7). Briefly, rats in the non-abstinent group were maintained on a continuous 2BC drinking protocol as reported previously(Ren *et al.*, 2023). Throughout the experiment, animals were given ad libitum access to 20% (v/v) ethanol solution and tap water, with no abstinence period imposed. Bottle positions were rotated daily to avoid side preference. All rats in this group were euthanized on Monday morning at week 9. Brain tissues were harvested and stored for subsequent quantitative real-time polymerase chain reaction (qRT-PCR) assays. The full experimental timeline is illustrated in Supplementary Figure 7A.

One-way ANOVA indicated a significant difference in *Cbr4* expression across the Post-EtOH, Naïve and Non-abstinent groups (F_2,27_ = 48.63, ##p < 0.01). Post-hoc analysis using Tukey’s HSD test further demonstrated that the mRNA expression of Cbr4 in the central amygdala (CeA) was significantly upregulated in the alcohol withdrawal group when compared with the Naïve and non-abstinent groups (**p < 0.01). There was no statistically significant difference in *Cbr4* expression between the Naïve rats and the Non-abstinent rats. Taken together, these data suggest that the increased expression of *Cbr4* in the CeA is specifically induced by alcohol withdrawal, rather than chronic ethanol exposure itself.
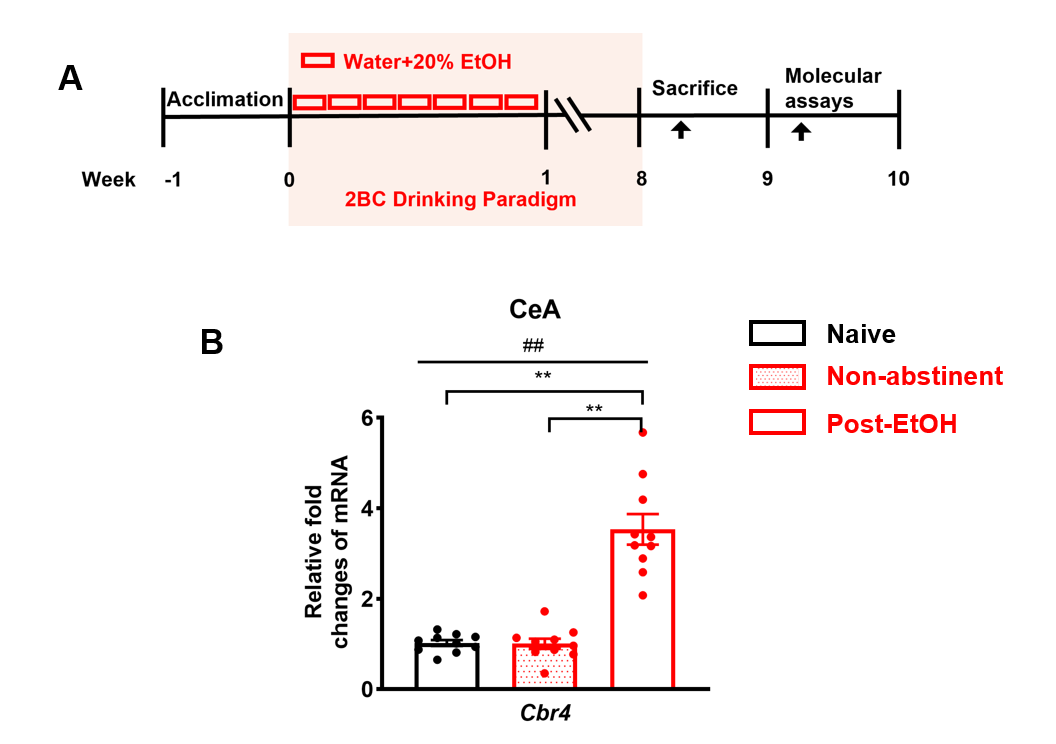


Supplementary Figure 7

Chronic alcohol drinking changes CeA *Cbr4* expression

Panel A is a schematic that shows alcohol or water in the continuous 2BC paradigm for 8 weeks. (All animal modeling and behavioral assessments were completed prior to initial manuscript submission. During the revision period, only quantitative detection and statistical analysis of *Cbr4* expression in the CeA were performed on pre-collected tissue samples)

Panel B summarizes the relative mRNA fold change of *Cbr4* in the CeA. One-way ANOVA revealed a significant difference in mean values among the groups (F_2,27_ = 48.63, ^##^*p* < 0.01). Subsequently, Tukey’s Honestly Significant Difference (Tukey’s HSD) post hoc test was used for pairwise comparisons of *Cbr4* mRNA expression levels in the CeA among the Naïve, Post-EtOH, and Non-abstinent groups (***p* < 0.01). n=10 rats/group. All data are shown as mean ± SEM.

2.Tables

Primer Sequences for Reverse Transcription-Quantitative Polymerase Chain Reaction

*Table 1. Reverse transcription-quantitative polymerase chain reaction primers*

| Primers | Sequences | Target Gene |
| --- | --- | --- |
| Acaa2-R | TGGCGCTGCTACGAGGT | Acaa2 |
| Acaa2-R | TCAGGCTGTGTTCTGGATGAT |  |
| Aldh1a1-F | ATGTCTTCCCCTGCACAGC | *Aldh1a1* |
| Aldh1a1-R | TTAGGAGTTCTTCTGAGATATTTTCATTG |  |
| Cbr4-F | ATGGACAAAGTCTGTGCGGT | *Cbr4* |
| Cbr4-R | TTAGGCAGTGAGCTGTAATCC |  |
| Itgal-F | ATGAGTTTCCGGATCCCTG | *Itgal* |
| Itgal-R | TTAGTCCTTGTCACTCTCACG |  |
| Mecr-F | ATGTTGGTCAGCCGGCGA | *Mecr* |
| Mecr-R | TCACATAGTGAGAATCTGCTTC |  |
| Ncaph-F | ATGAAAATCCCACGCTCAG | *Ncaph* |
| Ncaph-R | TCAGTCCCCTTGCATTACC |  |
| Tp53i13-F | ATGGTTCCTCCTCCTCCAC | *Tp53i13* |
| Tp53i13-R | TCAGTCCGAGCTCTCCGAGT |  |
| Orc4-F | ATGAGCAATCGTAAAAGCAAGAATAAC | Orc4 |
| Orc4-R | TCACAGCCAGCTGAGCGA |  |
|  |  |  |

3.Methods and materials

3.1Open Field Test (OFT)

OFT was performed following a standard protocol. Test apparatus: a blue Plexiglas square box (100×100×50 cm, L×W×H), with the floor divided into a central area (70×70 cm) and a peripheral area. Each rat was gently placed in the same corner of the box, and its behavior was video-recorded for 5 min using a camera mounted above the apparatus.

Behavioral data were analyzed using TopScan™ 3.0 (Clever Sys., USA) to quantify total distance traveled, central area distance, and inner/total distance ratio. Reduced central exploration (lower central distance and ratio) indicates increased anxiety-like behavior. After each test, the box was thoroughly cleaned with 15% ethanol to eliminate residual odors.

3.2 Elevated Plus Maze Test (EPM)

EPM is another well-established assay for assessing rodent anxiety-like behaviors. Apparatus: an elevated cross maze (50 cm above ground) with four arms (two open arms: 50×10 cm; two closed arms: 50×10 cm with 40 cm high opaque walls) and a central platform (10×10 cm).

Each rat was placed on the central platform facing an open arm and allowed to explore the maze for 5 min. Behaviors were video-recorded to count entries into open/closed arms (defined as all four paws entering an arm) and record time spent in each arm.

Increased open arm entries and time indicate reduced anxiety, while decreased exploration reflects enhanced anxiety-like behaviors. The maze was cleaned with 15% ethanol after each test to remove residual scents.

3.3 RNA Isolation, cDNA Synthesis, and qPCR Quantification

Total RNA was extracted from the CeA tissues of male Long-Evans rats (n=10/group) using TRIzol® reagent (Invitrogen, Thermo Fisher Scientific, Inc., Waltham, MA, USA), with all operations conducted on ice to prevent RNA degradation. RNA quality was quantified using a spectrophotometer, ensuring the absorbance ratio at 260-280 nm ranged from 1.8 to 2.0.

Reverse transcription was performed using the FastKing Kit (with gDNase) (Tiangen Biotech, Beijing, China), using 100 ng of total RNA as the template. This reaction was conducted in two sequential steps: first, 5×gDNA buffer was added, and the mixture was incubated at 42°C for 3 min to eliminate potential genomic DNA contamination; subsequently, 10×King RT buffer, Fast RT Enzyme Mix, and FQ-RT Primer Mix were supplemented, followed by incubation at 42°C for 15 min to complete reverse transcription and a subsequent incubation at 95°C for 3 min to inactivate the reverse transcriptase. For qPCR analysis, 60 ng of cDNA from each sample was used as the template.

qPCR was performed using SuperReal Premix Plus (SYBR Green) reagents (Tiangen) on the Bio-Rad CFX96 qPCR System (Hercules, CA, USA). The thermocycling conditions were set as follows: 95°C for 15 min (pre-denaturation); 95°C for 10 sec and 60°C for 30 sec (40 cycles, PCR stage); 95°C for 15 sec, 60°C for 1 min, and 95°C for 1 sec (melt curve stage to verify product specificity).

The relative changes in mRNA transcription levels were calculated using the comparative cycle threshold (2-ΔΔCt) method. Glyceraldehyde-3-phosphate dehydrogenase (GAPDH) was used as the endogenous control gene, and the ratios of mRNA expression of target genes to GAPDH were compared between groups. Each sample was independently assayed in triplicate, and the mean value of the three replicates was defined as the relative expression level of the target gene in the corresponding sample.

Reference：

Ren, Z. *et al.* (2023) “LPA1 receptors in the lateral habenula regulate negative affective states associated with alcohol withdrawal,” *Neuropsychopharmacology: Official Publication of the American College of Neuropsychopharmacology*, 48(11), pp. 1567–1578. Available at: https://doi.org/10.1038/s41386-023-01582-8.
